# Demyelination in Mild Cognitive Impairment Suggests Progression Path to Alzheimer’s Disease

**DOI:** 10.1371/journal.pone.0072759

**Published:** 2013-08-30

**Authors:** Cristian Carmeli, Alessia Donati, Valérie Antille, Dragana Viceic, Joseph Ghika, Armin von Gunten, Stephanie Clarke, Reto Meuli, Richard S. Frackowiak, Maria G. Knyazeva

**Affiliations:** 1 LREN, Department of Clinical Neurosciences, Centre Hospitalier Universitaire Vaudois (CHUV), and University of Lausanne, Lausanne, Switzerland; 2 Service of Old Age Psychiatry, Department of Psychiatry, Centre Hospitalier Universitaire Vaudois (CHUV) and University of Lausanne, Switzerland; 3 Neuropsychology and Neurorehabilitation Service, Department of Clinical Neurosciences, Centre Hospitalier Universitaire Vaudois (CHUV) and University of Lausanne, Lausanne, Switzerland; 4 Neurology Service, Department of Clinical Neurosciences, Centre Hospitalier Universitaire Vaudois (CHUV) and University of Lausanne, Lausanne, Switzerland; 5 CIBM (Centre d’Imagérie Biomédicale), CHUV unit, Lausanne, Switzerland; 6 Department of Radiology, Centre Hospitalier Universitaire Vaudois (CHUV), and University of Lausanne, Lausanne, Switzerland; Beijing Normal University, Beijing, China

## Abstract

The preclinical Alzheimer's disease (AD) - amnestic mild cognitive impairment (MCI) - is manifested by phenotypes classified into exclusively memory (single-domain) MCI (sMCI) and multiple-domain MCI (mMCI). We suggest that typical MCI-to-AD progression occurs through the sMCI-to-mMCI sequence as a result of the extension of initial pathological processes. To support this hypothesis, we assess myelin content with a Magnetization Transfer Ratio (MTR) in 21 sMCI and 21 mMCI patients and in 42 age-, sex-, and education-matched controls. A conjunction analysis revealed MTR reduction shared by sMCI and mMCI groups in the medial temporal lobe and posterior structures including white matter (WM: splenium, posterior corona radiata) and gray matter (GM: hippocampus; parahippocampal and lingual gyri). A disjunction analysis showed the spread of demyelination to prefrontal WM and insula GM in executive mMCI. Our findings suggest that demyelination starts in the structures affected by neurofibrillary pathology; its presence correlates with the clinical picture and indicates the method of MCI-to-AD progression. In vivo staging of preclinical AD can be developed in terms of WM/GM demyelination.

## Introduction

In the last decade, mild cognitive impairment (MCI) has received special attention as a likely precursor of Alzheimer’s disease (AD). MCI is classified into several phenotypic subtypes, of which the amnestic (aMCI) is the most strongly linked to AD [1], [2], [3]. Within this category, there are a number of forms varying from an exclusive memory deficit (single-domain aMCI or sMCI hereafter) to its combinations with inadequate performance in other cognitive domain(s) (multiple-domain aMCI or mMCI hereafter). The typical progression to AD starts with memory and learning problems, followed by deficits in executive functions, language, and praxis; ultimately, the entire cognitive sphere is affected [4], [5], [6]. Consistent with this dynamic, sMCI shows a higher incidence of improvement and carries a lower risk of conversion to AD than does mMCI [7], [8], [9], [10].

Yet this cognition-based classification is diagnostically unstable and has limited predictive validity in the general population [10], [11], [12]. Better performance is to be expected from an improved understanding of the neurobiological substrates of clinical aMCI phenotypes. To this end, neuroimaging of gray matter (GM) has been used. Volumetric voxel-based morphometry (VBM) studies have shown GM atrophy to be mainly restricted to the medial temporal lobe (MTL) in sMCI in contrast to a more extensive pattern of atrophy in mMCI individuals [13], [14]. A more recent VBM study of an aMCI population failed to show GM atrophy in sMCI, but did demonstrate atrophy in the hippocampus and temporal and frontal cortices in mMCI subjects [15]. Thus, the limited literature reporting on GM atrophy in aMCI suggests that individuals with this clinical syndrome may pass through different stages in progressing to AD [16].

When cortical cells die, their axons degenerate, so there must be destruction of myelin sheaths and failure of inter-regional cortical connectivity. In AD, white matter (WM) damage spreads in a relatively predictable pattern, with the latest structures to mature being the first to degenerate [16], [17], [18], [19]. The accompanying failure of cerebral connectivity interferes with cognition [20], [21], [22], so that extension of demyelination is to be expected in the progression from sMCI to mMCI.

To estimate the structural integrity of cerebral connections in aMCI, diffusion tensor imaging (DTI) has been used widely [23], [24], [25], [26], [27], reviewed in [28], [29]. The WM abnormalities are consistently found in posterior regions including the MTL, the splenium of the corpus callosum (CC), the posterior cingulum, and parietal WM, *i.e.*, in regions typically affected by AD. Some authors emphasize that among DTI parameters, radial diffusivity, associated with myelin breakdown, is particularly sensitive to aging and MCI-related WM changes [30], [31], [27]. Alternatively, myelin breakdown can be estimated by magnetization transfer imaging (MTI), which provides a myelin-based contrast independent of the spatial organization of WM fibers [32]. MTI permits an accurate evaluation of demyelination in aging and in populations with myelination abnormalities [33], [34], [22]. Animal models and postmortem studies of patients with AD or multiple sclerosis document that both DTI and MTI correlate with demyelination and axonal loss [33], [35], [36]. For whole brain mapping, MTI is an optimal choice, because it does not depend on fiber orientation and preserves accuracy throughout the entire brain space. Thus far, neither DTI nor MTI results have been compared between aMCI subtypes.

**Figure 1:**
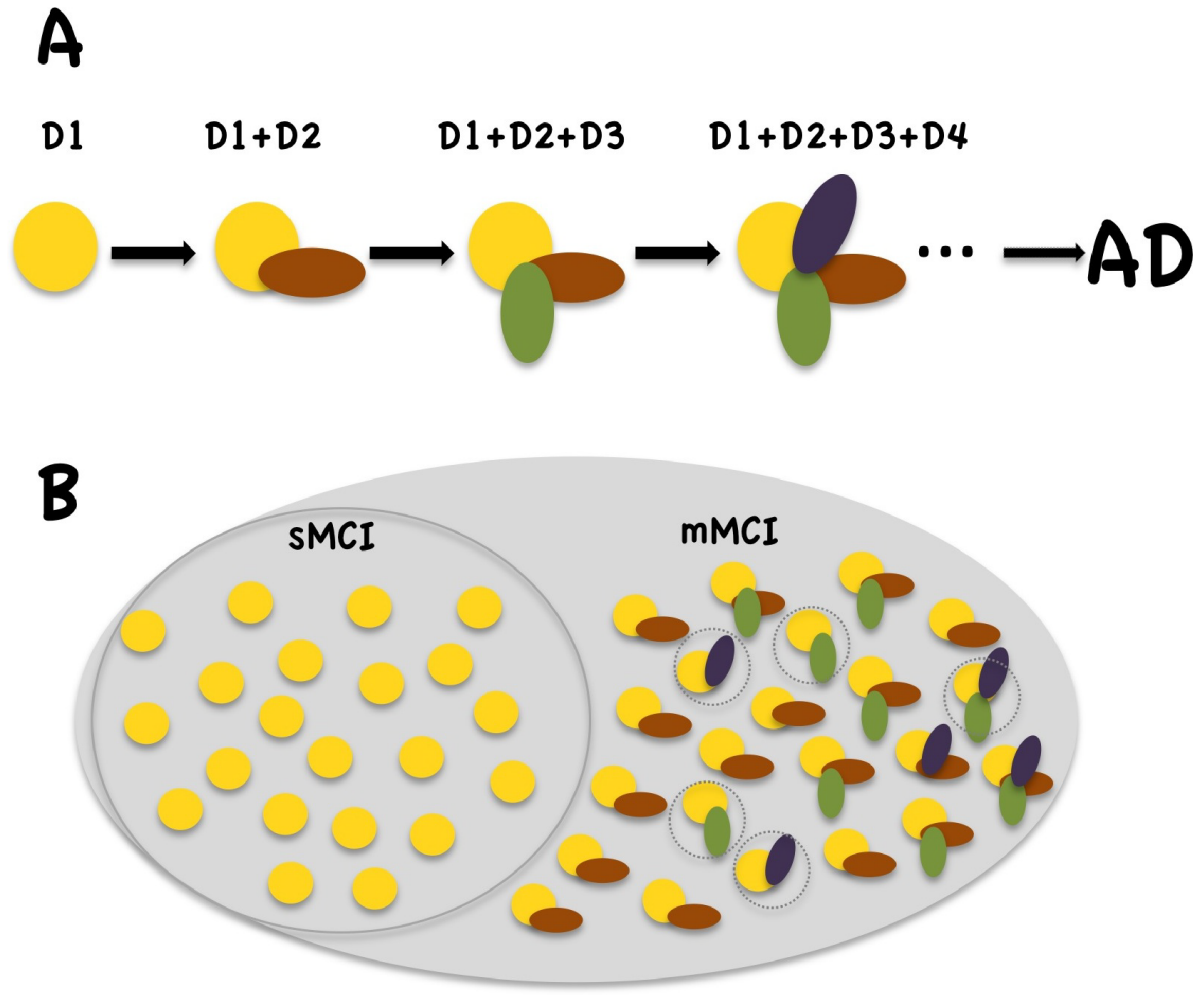
Progression of amnestic mild cognitive impairment to Alzheimer’s disease. **A** represents schematically the typical progression from amnestic MCI to AD manifested at the clinical level by a uniformly ordered sequence of cognitive phenotypes with deficits in domains D1, D2, D3, …, which accumulate in that order beginning with the initially affected memory domain (D1). Ideally, all multiple-domain MCI (mMCI) individuals will show at least D1 (memory deficit designated with yellow circle) and, D2, the next affected domain. If affected cognitive domains accumulate due to the spreading of an initial local pathological process, the spreading beyond the sMCI stage can be deduced from a comparison between mMCI and single-domain MCI (sMCI) groups. However, as shown in **B**, some mMCI subjects manifest atypical combinations of affected cognitive domains, suggesting causes other than prodromal AD. **B** depicts a sample of 42 aMCI individuals, 21 of whom (encircled on the left) have sMCI and 21 have mMCI. In the latter group, subjects with atypical clinical phenotypes are in dashed circles. According to the hypothesis of “single source–common path” for typical aMCI-to-AD progression, a contrast between sMCI and the largest mMCI subgroup with two common deficits (here D1+ D2) should target the next stage along the progression path.

In this study, we therefore analyze the myelination state across different aMCI subtypes. The analysis framework is based on the hypothesis that demyelination in the progression from aMCI to AD originates from a single source and follows a common scenario. At the clinical level, this is manifested by a uniformly ordered sequence of cumulative cognitive deficits, beginning with an initially affected cognitive domain [37], [38]. Based on structure-function relationships, we take the multiplicity of affected cognitive domains to be the result of this spread of an initial local pathological process in the brain. If the hypothesis of “single source–common path” is correct for *typical* aMCI-to-AD progression, a common region of demyelination should exist in all aMCI subtypes. The subsequent spread of demyelination can be deduced from a comparison between mMCI and sMCI subjects (Fig. 1). It should be mentioned that by suggesting *typical* aMCI-to-AD progression path, the hypothesis implies neither equivalence between MCI and AD, nor strict uniformity of the progression path.

Predictions from the “single source–common path” hypothesis include *(1)* a *conjoint* region of demyelination across the entire aMCI sample and *(2)* a *differential pattern* of regional demyelination in mMCI that represents its spread along a typical path from aMCI to mMCI. To test these predictions formally, we use conjunction/disjunction MTI analysis of both subcortical WM and intracortical myelin.

## Methods

### 2.1. Ethics Statement

All investigative methods and procedures applied in this study conform to the Declaration of Helsinki (1964) of the World Medical Association concerning human experimentation. The authors state that they have obtained approval from the local Ethics Committee of Lausanne University. All study subjects gave written informed consent at the time of enrollment for the research project 320030-127538/1 “Cerebral network function in neurodegeneration: A multimodal approach” funded by Swiss National Science Foundation. All potential participants who declined to participate or otherwise did not participate were eligible for treatment and were not disadvantaged in any other way by not participating in the study. The consent capacity of MCI subjects and controls has been determined by the physician (JG or AvG) and neuropsychologist (VA or AD) based on the interview of a potential subject (supplemented, when possible, by that of an informant in the case of MCI) and confirmed by a comprehensive neuropsychological assessment of the subject's cognitive abilities (see Section 2.3.). All controls and MCI subjects from the sample reported here were able to understand the essential information about the research project and to make and communicate a valid consent.

### 2.2. MCI and Control Subjects

Here we present cross-sectional results of a larger study that includes a follow-up analysis of demyelination in the sample screened for clinical MCI syndrome. Considering demyelination pattern as a potential biomarker for AD pathology, we avoided restricting our clinical MCI sample by introducing other biomarkers to qualify underlying AD pathology. Forty-two subjects (50 years and older) diagnosed with MCI were recruited from the Memory Clinic of the Neurology service, the Old Age Psychiatry service, and Neuropsychology and Neurorehabilitation service of the CHUV in Lausanne, and 42 age-, gender-, and education-matched control subjects, selected from partners, caregivers, and family members, were enrolled (Table 1).

**Table 1:** Demographic and clinical characteristics of MCI and control subjects.

Feature	sMCI	mMCI	Executive mMCI	Controls	Statistical comparisons		
**# of subjects**	21	21	16	42	―		
**Gender M/W**	8/13	8/13	8/8	16/26	*NS	**NS	***NS
**Age in years**	70.4±1.6	70.8±1.7	72.9±2.0	69.4±1.1	*NS	**NS	***NS
**Education**	#1/#2/#3 2/15/4	#1/#2/#3 3/11/7	#1/#2/#3 3/8/5	#1/#2/#3 5/23/14	*NS	**NS	***NS
**MMSE**	28±.3	27.3±.4	27.2±.4	29.0±.2	*NS	** *P*<.005	***P<.0002
**BADL**	6±0	6±0	6±0	6±0	*NS	**NS	***NS
**IADL**	8±0	8±0	8±0	8±0	*NS	**NS	***NS
**HAD**	8.7±1.1	9.8±1.7	9.9±1.9	7.8±.8	*NS	**NS	***NS

Columns present group characteristics (mean ± standard error of the mean). “M” stands for men, “W,” for women. Educational status was determined by 3 categories: 1 – primary/secondary school without or with short (<3 years) professional training; 2 – primary/secondary school with professional training (>3 years); 3– high school and tertiary education.

* stands for the contrast *sMCI>mMCI*;

** for *controls>sMCI*, and ***controls>mMCI. “NS” stands for “not significant” (*P*≥.05, non-parametric ANOVA with three levels and χ^2^ test with Bonferroni correction for the three pairs of comparisons). We applied 10000 permutations to estimate the distributions of the t-contrasts. There were no significant differences between any aMCI subgroups for the parameters presented in the table.

The clinical diagnosis of MCI was based on the criteria proposed by [1] and confirmed in the recent recommendations of the National Institute on Aging and Alzheimer’s Association workgroups [39]. The selected aMCI groups were also characterized using MRI-based volumetric measurements of the hippocampus as a structural biomarker of AD (see Section 3.1.). MCI was diagnosed in individuals with mild cognitive decline (as corroborated by an informant), and confirmed by a neuropsychological examination including the Mini-Mental State Examination (MMSE). Only individuals who did not satisfy the NINCDS–ADRDA criteria for AD or other types of dementia were selected for MCI diagnosis [40]. Specifically, MMSE scores between 24 and 26 for low-level education and between 24 and 28 for high-level education would qualify. Our MCI sample included single-domain amnestic MCI (sMCI, 21 subjects) and multiple-domain amnestic MCI (mMCI, 21 subjects), determined by neuropsychological testing as described in Section 2.2.

Clinical laboratory investigations and diagnostic neuroimaging (CT or MRI and Metrizamide SPECT) were performed to exclude subjects with other causes of cognitive deficit (stroke, expanding processes, etc.), severe physical illness, psychiatric or other neurological disorders potentially associated with cognitive dysfunction, and non-AD dementing conditions (fronto-temporal dementia, dementia associated with Parkinsonism, Lewy body disease, pure vascular or prion associated dementia, *etc*.). Alcohol/drug abuse and regular use of neuroleptics, antidepressants with anticholinergic action, benzodiazepines, stimulants, or β-blockers were also exclusion criteria.

To confirm the absence of psychoactive drug use or other diseases that interfere with cognitive functions, potential control subjects underwent a brief clinical interview including the MMSE and a brain MRI. Only individuals with no cognitive complaints, normal activities of daily living assessed by the BADL and IADL scales [41], and an MMSE score ≥26 for low and ≥28 for high level of education were accepted as controls.

### 2.3. Neuropsychological Testing

All participants were administered an extended battery of neuropsychological tests, comprehensively covering 5 cognitive domains (memory, executive functions, language, praxis, and gnosias). *Episodic memory* was assessed by a verbal RI-48 and/or RL/RI 16-item task [42] and by visuo-spatial tasks from the Doors test (the Doors and People battery [43]). *Short-term memory* was assessed by a verbal digit span task [44], a visuo-spatial span task [45], and the verbal reverse digit span task from the WAIS-III [46].

To assess *executive functions,* we used three tests proposed by the GREFEX manual [47]: a verbal fluency task (categorical and literal fluency in 2 minutes), a flexibility task (the Trail Making Test part 2), and an inhibition task (the Stroop test). *Language* was assessed by an object denomination (Lexis test, [48]). We assessed *praxis* by using a brief clinical scale for gestural abilities of the upper limbs (the Bbep, [49]). *Gnosias* were assessed by a test of discrimination of overlapping figures (subtest of the BEN [50]).

Testing was completed by the Mini-Mental State Examination (MMSE, [51]) in French adaptation [52]. The BADL/IADL scales [41] were used to assess the impact of deficits on daily living activities, and a depression and anxiety self-assessment was done with the HAD scale [53], to exclude major depression and also to quantify subtle fluctuations of mood among serial evaluations.

An individual was diagnosed with amnestic MCI if (s)he met the following three criteria: 1) had at least one score on an episodic memory task of ≥1.5 standard deviation (SD) below normative values; 2) lost ≤2 points on the BADL/IADL scale; 3) had 24≤MMSE≤26 for a low level and 24≤MMSE≤28 for a high level of education.

The distinction between sMCI and mMCI was based on the summarized scores of all tests. Individuals with a score ≥1.5 SD below the respective normative value in at least one episodic memory test but normal scores in other domains were classified as sMCI. Alternatively, individuals with scores below the 1.5 SD in at least one episodic memory test in association with impaired performance in at least one other domain were considered mMCI.

### 2.4. Magnetic Resonance Imaging

All MCI and control subjects were scanned on a 3 Tesla Siemens Trio scanner with a 32-channel head coil. A high-resolution T1-weighted two inversion-contrast Magnetization Prepared Rapid Gradient Echo (MP2RAGE) sequence was acquired (TR = 5000 ms, TE = 2.84 ms, FoV = 256×240×160, voxel size = 1×1×1.2 mm^3^). The MP2RAGE acquisition corrects B1 field inhomogeneities for T1-mapping [54]. MTI was acquired through Multiple echo Fast Low Angle SHot (FLASH) Magnetic Resonance Imaging (TR = 48ms, TEs = 2.33, 5.3, 8.2, 11.2, 14.2, 17.1, 20.1, 23.1 ms; FoV = 120×128×96, voxel size = 2×2×2 mm^3^) as described in [55]. The protocol included running this gradient-echo sequence twice: first with and then without a radio-frequency (RF) pulse. Since the effect of MT depends mainly on restricting the mobility of water protons by macromolecules, a large contribution comes from myelin [32]. Water protons bound to macromolecules exhibit a much broader absorption spectrum than free-water protons when saturated using an off-resonance RF pulse. Consequently, MT will be weaker with the RF pulse applied than without. The resulting contrast allows for quantification of the amount of myelin with the MT ratio (MTR) [32].

We averaged multiple (8 TEs) gradient echo MTI acquisitions to increase signal-to-noise ratio [56]. These images were co-registered to the high-resolution MP2RAGE T1w scans. We calculated the MTR in every voxel (vol = 8 mm^3^) using the expression
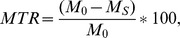
where *M*_0_ stands for the intensity of a voxel without saturation and *M*_S_ with saturation. By definition, the ratio indicates the percentage loss of signal intensity attributable to the MT effect. Since this effect is mainly dependent on myelin [32] a decrease in MTR values is considered to be a sign of demyelination.

For population analysis, the MTR images of each subject were non-linearly transformed to standard MNI space using the diffeomorphic registration algorithm (DARTEL; [57]) implemented in SPM8 (www.fil.ion.ucl.ac.uk/spm). The applied DARTEL flow field was previously estimated by the simultaneous inter-subject alignment of gray matter (GM) and white matter (WM) maps, resulting from the segmentation of the T1-weighted images into GM, WM, and cerebrospinal fluid (CSF) [58]. To enhance the specificity of tissue class and to account for the partial volume contribution to each voxel, a combined weighting/smoothing procedure was implemented [59]. Specifically, for each subject, the MTR map normalized to MNI space was, first, multiplied by the normalized tissue maps (GM, WM), second, modulated by the Jacobian determinant of the DARTEL deformation field, and third, smoothed with an isotropic Gaussian kernel of 4 mm full-width at half-maximum (FWHM). Finally, we divided each of the two MTR maps (GM, WM) by the smoothed, modulated, and weighted tissue map.

### 2.5. Statistical Design and Inference

To exploit the commonality and heterogeneity of the amnestic MCI cohort, we implemented a conjunction/disjunction inferential approach [60] through a one-way statistical design with three levels: sMCI, mMCI, and controls. The logical “AND” (conjunction) of the two contrasts *controls>sMCI* and *controls*>*mMCI* shows common structural changes for sMCI and mMCI individuals, i.e., core features of the entire MCI group. The structural changes specific to sMCI or mMCI subjects are revealed by a contrast between these groups (i.e., the logical “NOT” – disjunction).

The voxel-based statistical analysis was embedded in the General Linear Model framework of SPM8. To avoid contamination by misclassified voxels, we masked the MTR images by corresponding tissue maps (GM or WM) thresholded at *P* = .2. This threshold ensures the inclusion of voxels most likely representing the tissue of interest.

Statistical inference was based on the threshold-free cluster enhancement (TFCE) statistic [61]. Contrary to cluster inference approaches based on random field theory, the TFCE method allows cluster-level inference without applying an arbitrary cluster-forming threshold (*th_c_*) and it is robust to non-stationary smoothness in brain images [62]. TFCE avoids an arbitrary choice of a single *th_c_* value by gathering cluster size information within the range of possible *th_c_* values. The method provides a voxel-wise image, in which each voxel value represents the cumulative cluster-like evidence within its spatial neighborhood over the range of cluster-forming thresholds. To assay inference at the family-wise error (FWE) significance level of 95%, the null distribution of the maximum TFCE value across the volumes of interest (GM, WM) was estimated through 10000 random re-labelings of group membership (aMCI, mMCI, and controls), and the 95th percentile of such a null distribution was applied to threshold the actual TFCE images. The computations were performed with a TFCE toolbox for Matlab (http://dbm.neuro.uni-jena.de/tfce/).

We analyzed the association between MTR maps and neuropsychological scores of interest with multiple linear regression models. In all the models, age, gender, and level of education were included as covariates in order to adjust for their impact. In one model, the MTR maps were regressed with scores from the delayed cued recall of the RI-48 test, available for 69 subjects (27 MCI and 42 controls) (Table S1). To increase specificity of changes associated with the delayed recall, we included the immediate recall score as a covariate. Statistics were performed as described for the conjunction design.

We estimated the effect size of the cluster-wise conjunction/disjunction effects through the Mahalanobis distance *D*, a multivariate generalization of Cohen's univariate effect size. Specifically, *D* represents the standardized difference between two groups along the discriminating axis (here, along MTR); for example, *D = *1 means that the two group centroids are one standard deviation apart along the MTR axis. In the formula
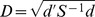

*d* is the vector of univariate differences and *S* is the pooled covariance matrix. Since there were more voxels than samples at each cluster of interest, we computed a regularized estimation of *S* [63]. Further, as for the magnitude of the effects, we estimated the precision of *D* (that is, confidence intervals) through 5000 bootstrap samples [64].

For anatomical labeling we used the AAL atlas [65] and the ICBM DTI-81 atlas [66].

## Results

### 3.1. Main Neuropsychological and Structural Characteristics of aMCI Subtypes

Consistent with our diagnostic criteria, both sMCI and mMCI groups significantly differed from controls on the MMSE scores (*P*<.05, Bonferroni corrected) and in episodic memory performance (both immediate and delayed cued recall in RI-48) (*P*<.05, Bonferroni corrected; Tables 1 and S1). However, these scores did not differ between aMCI sub-groups. In contrast, executive functions were impaired not only in sMCI and mMCI compared to controls (*P*<.05, Bonferroni corrected), but in mMCI compared to sMCI (*P*<.01, Bonferroni corrected; Table S2). The BADL/IADL and HAD scales did not differ among the three groups.

**Figure 2:**
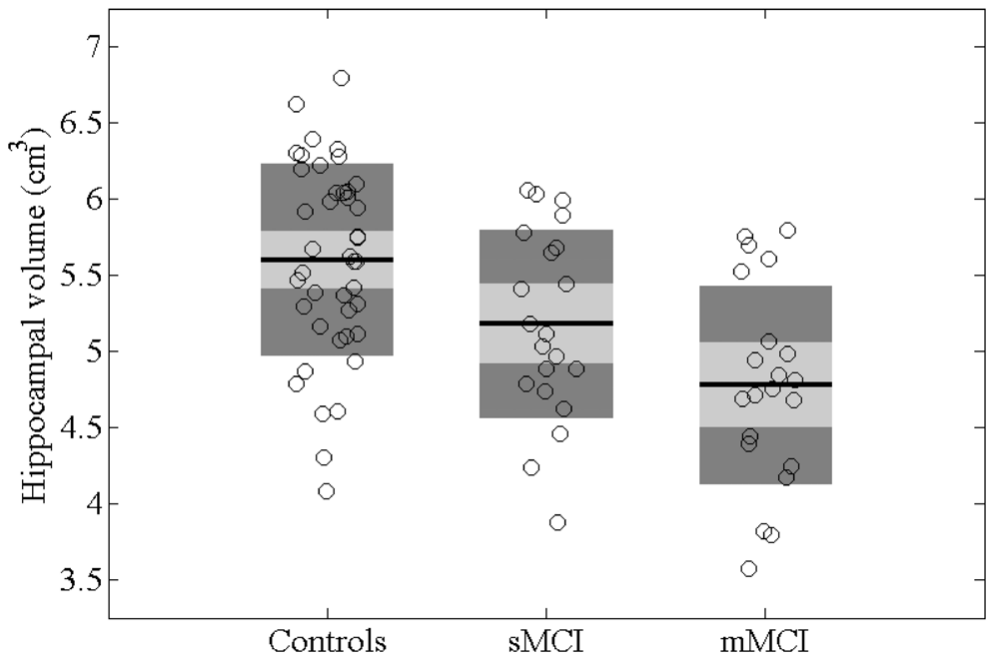
Hippocampal volume in control subjects and in subjects with amnestic mild cognitive impairment. The total (left hemisphere+right hemisphere) volume of the hippocampus is shown for the control, sMCI and mMCI groups. For each group, the estimated individual values are shown with empty black-bordered circles. The black lines represent the group mean, the light gray boxes represent the interval spanned by Mean ±1 SD, and the dark gray boxes, Mean ±1.96 SD. Both between-group contrasts (*sMCI<Controls* and *mMCI<Controls*) are significant (for details see Section Results).

Hippocampal atrophy is an in vivo surrogate indicator of the neurodegenerative aspect of AD pathology. To validate the condition of our amnestic MCI sample as a prodromal stage to AD, we additionally measured the hippocampal volume in all the participants of this study. The total volume of the hippocampus was estimated using SPM8 through the following steps. First, a mask of the two hippocampi in the MNI space was obtained using the AAL atlas [65]. This mask was projected into each subject’s space through the inverse deformation fields delivered by the unified segmentation routine of SPM8. Finally, the individual volume of the hippocampus corresponded to the amount of voxels extracted via the subject-specific mask. The estimated hippocampal volumes are shown in Fig. 2. The values in our control group match those obtained in other cohorts with manual segmentation and/or automatized techniques [67], [68]. We evaluated the between-group differences within the framework of the General Linear Model, including the total intracranial volume as a covariate. The distribution of the contrasts of interest was estimated through 10′000 permutations. In the sMCI group, the hippocampal volume was 8% lower than in control subjects at *P* ∼ 0.013, while in mMCI it was 15% lower at *P*<0.0001. The average volume reduction in our MCI sample is similar to 11–13% as previously demonstrated for MCI subjects [69].

### 3.2. Conjunction and Disjunction Analysis of Amnestic MCI

**Figure 3:**
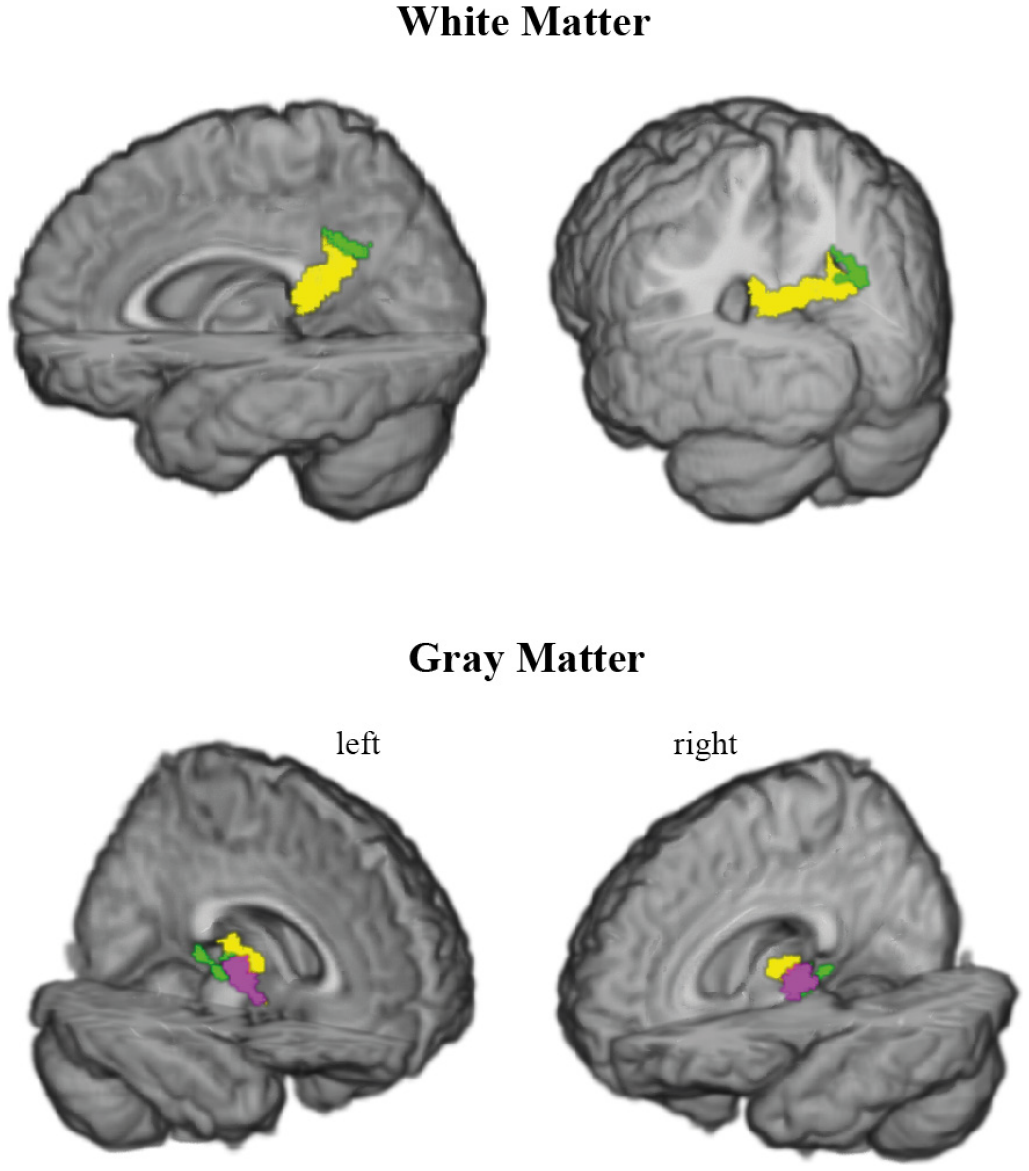
Conjunctive demyelination in sMCI and mMCI subjects. The conjunction effects are rendered in different colors corresponding to different anatomical structures. Here and hereafter, for presentation purposes, the SPM (*P*<0.05, FWE corrected) is overlaid on a single subject T1-weighted image rendered by means of the mricrogl software (http://www.mccauslandcenter.sc.edu/mricrogl/). The colored regions are projected on the shown brain section. The top row shows the conjunctive demyelination of the splenium (yellow) and posterior corona radiata (green) in the medial (left) and posterior (right) view. The bottom row shows the conjunctive demyelination of the hippocampus (yellow), the parahippocampal gyrus (violet), and the lingual gyrus (green) in the medial view (left and right hemispheres on the left and right, respectively).

Across the whole brain volume, we found an MTR decrease at *P*<.05 (FWE corrected) in the medial temporal lobe (MTL) and posterior parts of the brain bilaterally (Fig. 3, Table S3). The magnitude of the effect size in all significant clusters was reliably large (Table S4). Specifically, among the WM structures (Fig. 3, top row), the splenium of the CC in both hemispheres, and the right posterior corona radiata (specifically, fibers connecting the precuneus) showed reduced MTR values. Within the GM compartment, MTR was decreased in the hippocampus and in the parahippocampal and lingual gyri bilaterally, but more extensively in the left hemisphere, as well as in the thalamus and fusiform gyrus (Fig. 3, bottom row; Table S3).

We found no group-specific clusters differentiating the sMCI group from the entire mMCI group in any of the two compartments. Given that some individuals might have had aMCI due to other than prodromal AD causes (Fig. 1), an alternative contrast that targets the next stage along the progression to AD is that between sMCI and the largest homogenous mMCI subgroup *in the population at large*.

**Figure 4:**
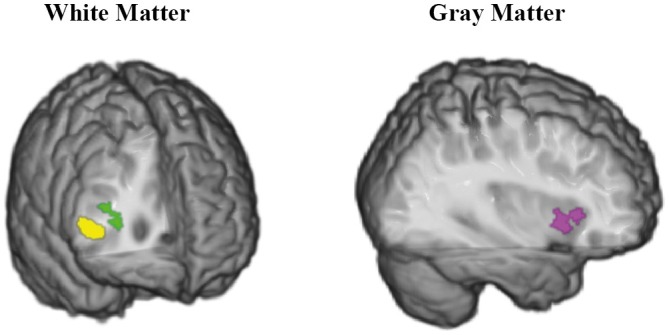
Disjunctive demyelination in mMCI with executive dysfunction compared to sMCI. The top row shows the disjunctive demyelination in the white matter of the pars triangularis of the right inferior frontal gyrus (yellow) and of the right middle frontal gyrus (green) in the coronal plane. The bottom row demonstrates the disjunctive demyelination of the right insula (violet) in the sagittal plane. For other designations see Fig. 3.

In our sample, the most numerous category of mMCI subjects (16 individuals) consisted of those with executive dysfunctions. Individuals were considered executive mMCI if they had scores below the 1.5 SD in at least one episodic memory test and one of the 3 executive tests (see Section 2.3.). In so doing, we also included patients with additional impairments in other domains. A *post-hoc* disjunction analysis showed a significant (*P*<.05, FWE corrected) MTR decrease in executive mMCI compared to the sMCI group in the right prefrontal WM and GM (Fig. 4, Table S3). Specifically, the disjunctive clusters spanned the WM below the pars triangularis of the inferior frontal gyrus and the middle frontal gyrus. In the GM, a disjunctive cluster was located in the insula. The magnitudes of effect size were large in both compartments (1.1–4.3 at 95% confidence intervals).

### 3.3. Classification of Amnestic MCI Subjects

To evaluate how well MTR maps discriminated between sMCI, executive mMCI, and controls, we applied a classification algorithm based on a combined feature selection and linear discriminant analysis (LDA) approach [70] (for details see Text S1). We assessed the accuracy of the three binary classifications *controls vs. sMCI*, *sMCI vs. executive mMCI,* and *controls vs. executive mMCI* (Table S5) by a leave-one-out cross-validation procedure. This showed that executive mMCI can be accurately predicted with respect to controls (accuracy of about 70% for both GM and WM compartments at *P*<.0001). The sMCI and executive mMCI groups were classified with an accuracy of 61%, at *P* = .055, while sMCI and controls were not distinguishable. There are potential technical improvements, including the use of quantitative MTI [71] and a reduction of feature dimensionality by brain parcellation that might increase the discriminative potential of MTI.

### 3.4. Voxel-based Morphometry

We repeated the above-described analysis of GM and WM volumes, computed by smoothing the modulated GM and WM T1-weighted images with a Gaussian kernel of 4 mm (FWHM). The statistical design included total intracranial volume as a nuisance covariate and the inference was again based on TFCE statistics (see Section 2.5.). No conjunction or disjunction (across sMCI, mMCI or executive mMCI) was found (*P*>.05, FWE corrected).

### 3.5. Multiple Linear Regression Analysis

**Figure 5:**
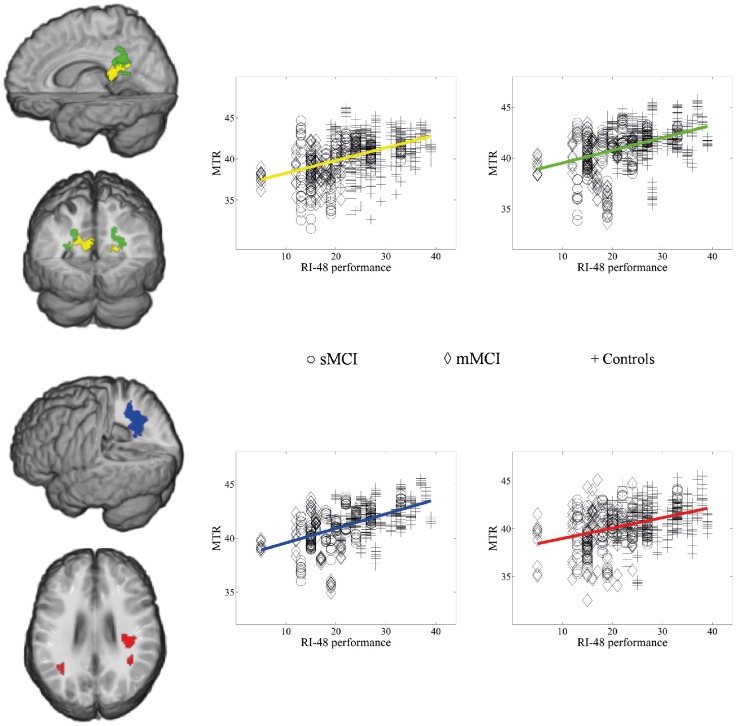
Statistical parametric map of dependence between episodic memory performance and demyelination. The brain regions with a voxel-wise positive linear dependence (*P*<.05, FWE corrected) between MTR and the delayed cued recall in the RI-48 test for 69 subjects are rendered in different colors corresponding to different anatomical structures. For each brain region, a scatterplot of the MTR values of ten voxels with the highest values of the TFCE statistic are shown for sMCI (circles), mMCI (diamonds), and controls (crosses). The two upper images on the left show the involved parts of the splenium (in yellow) and of the posterior corona radiata (in green). The average (over the selected ten voxels) slope of the linear dependence is .13 for the splenium and .12 for the posterior corona radiata, while the average R^2^ is .29 and .18, respectively. The third image (from the top) shows the dependence map in the white matter of the precuneus (in blue, the sagittal view). The average slope of the linear dependence is .16 and the average R^2^ is .29 for the precuneus. The bottom brain image shows the longitudinal superior fasciculus, for which the average linear slope is .11 and the average R^2^ is .14.

The delayed cued recall scores of the RI-48 test from 69 subjects explained the MTR reduction in projection, association, and commissural pathways including the splenium of the CC, the superior longitudinal fasciculus (predominantly in the right hemisphere), the posterior corona radiata, the posterior thalamic radiation, and the fornix of the limbic system bilaterally (Fig. 5 and S1, Table S6). The MTR in the inferior longitudinal and inferior fronto-occipital fasciculi and in the precuneus correlated with episodic memory performance only unilaterally. No correlations were found in GM.

## Discussion

### 4.1. Demyelination Shared by Single- and Multiple-domain aMCI

Regardless of subtype, the aMCI patients showed common sites of demyelination in both studied cerebral compartments. Specifically, the GM was bilaterally affected in the MTL (including the hippocampi and the parahippocampal gyri) and in the lingual gyri. This pattern of changes, together with the hippocampal atrophy documented in both aMCI groups, is typical for AD and therefore, consistent with the concept that in most patients aMCI represents prodromal AD. This observation supports the idea that significant intracortical reduction of myelin content is among the earliest events in the course of progression from aMCI to AD.

In a parallel VBM analysis of local brain tissue volumes, we failed to show any significant results in the framework of a conjunction analysis between sMCI and mMCI groups. Yet previously VBM-identified GM changes have been reported in aMCI subjects in the hippocampus and the parahippocampal, lingual, and fusiform gyri [72], [13], [14]. Although these findings converge on the regions implicated in AD, some of them have limited statistical validity, i.e., are uncorrected for multiple comparisons [13], [14], or may be due to the composition of the MCI group, which included a high proportion of incipient AD patients [72]. Recent VBM studies using rigorous statistical thresholds similar to those in our study failed to show GM reductions in sMCI [73] and mixed aMCI groups [74], [75]. In line with previous reports, there was GM atrophy in our mMCI group compared to matched controls. It was significant (P<.05, FWE corrected) in the hippocampus, amygdala, thalamus as well as in the parahippocampal, superior temporal, lingual, and fusiform gyri of both hemispheres.

Among WM structures, the extensive demyelination shared by the two aMCI subgroups was detected in the splenium and posterior corona radiata. These findings are in line with the few available DTI studies, which showed reduced integrity of the long-distance association WM tracts and of the corpus callosum in aMCI patients ( [76], [30], [77], for review see [16]). Moreover, our regression analysis linked the demyelination in these bundles and in the superior longitudinal fasciculus to episodic memory performance. In contrast, demyelination in the GM was not associated with the memory scores. Therefore, unlike in AD, early memory deficits in the preclinical stage reflect disconnection of long-distance pathways rather than local connectivity or GM atrophy, thus emphasizing the significance of functional and effective connectivity changes in preclinical AD [78], [79]. The changes of the intracortical myelin content, although quite extensive, do not correlate with clinical symptoms. These clinically silent alterations deserve further investigation.

### 4.2. Splenium of Corpus Callosum as Early Marker of AD

In aMCI, the splenium was the most affected bundle: about 40% of its voxels showed decreased MTR. In addition, splenial demyelination correlated with impaired episodic memory performance. Given the limited effects of CC sectioning on cognition (including memory) in split-brain patients [80], [81], [82], such a correlation suggests that splenial dysfunction is in some unique way an important feature of the AD pathological process.

The splenium carries fibers connecting the posteromedial cortices of the two hemispheres, including the MTL regions that are primarily affected in AD. They are linked with thin late-myelinating axons concentrated in the anterior part of the splenium [83], [84]. The callosally projecting neurons originate from and target supragranular (II, III) and infragranular (V) cortical layers [85], where neurofibrillary pathology and neuritic plaques are preferentially located in AD patients [86], [87].

Consistent with these results is a growing body of structural neuroimaging literature that supports the notion of early involvement of the splenium in AD-associated neurodegenerative processes. The reduced size of the splenium is found in sagittal MRI images in aMCI cases [88], [74]. Available DTI studies characterize the splenium in aMCI by decreased fractional anisotropy (FA) and/or increased mean diffusivity [89], [90], [91], [77], [92], although some groups have failed to confirm such changes [93], [73]. Zhuang and coauthors reported that the best discrimination (around 75%) between aMCI and controls is based on a combination of FA measures in the splenium and the crus of the fornix [26].

These properties of the splenium probably reflect structural features in the MTL associated with an increased risk of AD. In healthy aged APOE4 carriers, lower FA is described in the splenium [94], [95], [96]. Even in young people, gene variants that increase risk of AD in old age are associated with decreased FA in the splenium [97]. It is unlikely that these findings capture the first traces of degeneration in young people. Rather they indicate delayed myelination or a differential composition of low- and non-myelinated fibers in carriers of the AD-associated alleles. According to the “last myelinated, earliest demyelinated” hypothesis, such features could in turn predispose to AD.

Cumulatively, these data run counter to the recent proposal of Di Paola and colleagues that the posterior CC subregion is damaged solely by Wallerian degeneration in AD [98]. Instead, we suggest that both Wallerian degeneration and myelin breakdown, affecting later-myelinating CC subregions, contribute to the pathological alterations and dysfunction of the splenium, thus explaining its early involvement in aMCI-AD. Therefore, being well defined anatomically, comprehensively studied in human and animal models, and easily measurable with noninvasive neuroimaging techniques, the splenium of the CC represents an attractive candidate marker of Alzheimer risk and of progression to manifest disease. One issue that requires definition in future work is the nature of the temporal dynamics of *pre-MCI* changes in the splenium in the normal aging population.

### 4.3. Progression of Demyelination and MCI-AD Staging

In the mMCI group, the hippocampal volume was lower than in control subjects and in the sMCI group, suggesting progression of the underlying pathology. If the typical sequence of cognitive deficits in AD results from the spread of AD pathology throughout the brain, the largest proportion of mMCI individuals should present a memory deficit (characteristic of sMCI) and an additional common deficit. In our sample, the largest mMCI group (16 subjects) showed memory and executive dysfunctions.

Additional structural changes were expected in the executive mMCI patients compared to those with sMCI in prefrontal regions, known to be critical for such functions [99], [100]. Our disjunction analysis showed significant demyelination in the WM of the right inferior and middle frontal gyri and in the GM of the right insula, while there were no differences in GM or WM volumes. The fact that we and others [14] have failed to find a differential GM atrophy pattern in sMCI and executive mMCI suggests that early executive deficits result initially from a partial disconnection of the prefrontal cortex that precedes its atrophy, similarly to our sMCI patients with changes in the splenium of the CC in the absence of posteromedial cortical atrophy in sMCI. A recently shown reduction in glucose metabolism within distributed prefrontal networks, correlating with impairment of executive functions in MCI and AD patients [101], supports this interpretation. In the absence of atrophy, hypo-metabolism can result from partial isolation of a region due to disconnection.

To answer the question of whether a progression of demyelination into the prefrontal white matter associated with executive dysfunction captures the trajectory of AD progression, we must ask to what extent the predominance of executive mMCI subjects in our sample reflects the usual progression of pathological events. In short, is our sample biased? Community studies show that executive dysfunction is among the earliest signs of preclinical AD. In the Framingham cohort, which included more than two thousand individuals followed for 22 years, memory and abstract reasoning were the best predictors of AD, 10 and 5 years respectively, before the diagnosis [102]. Chen and coauthors tracked a non-demented cohort of more than 500 individuals for 10 years [103]. Those diagnosed with AD (68 individuals) showed earliest decline in memory and executive function 3.5 and 1.5 years before diagnosis, respectively. In the 187 participants of the Berlin aging study, attentional and executive tests predicted AD onset better than episodic memory tests 4 years before diagnosis [104]. Thus, the relative size of our executive mMCI group is consistent with representative community studies and corresponds to a common progression from sMCI. We find that it is characterized by demyelination spreading through the postero-medial region (in common with sMCI) and the inferior prefrontal regions.

The existing neurobiological staging of AD, based on the extension of neurofibrillary pathology, correlates with the deterioration of cognition on a coarse scale [105], [106]. According to this scheme, the MTL is the anatomical location of initial pathological changes in AD, which emerge years or even decades before the diagnosis [107], [108]. An *in vivo* staging of preclinical AD based on reliable neuropathological features is currently unavailable. Our findings suggest that demyelination, which reflects the loss of inter-regional cortico-cortical and cortico-subcortical connections, and is quantifiable by MRI, is a candidate feature that can be used ethically and non-invasively in life. Work must now be done to generalize the group results we report here to individual patient studies. As this has already happened in AD with VBM studies by means of machine learning based classification algorithms, this is not a far-fetched idea. Demyelination is initiated in the same structures that accumulate neurofibrillary pathology, it correlates with clinical features on a fine scale, and suggests a mechanism for the process of aMCI to AD progression. Therefore, we suggest that *in vivo* staging of preclinical AD by assessment of the spread of WM damage is a promising route for further research and development.

### 4.4. Limitations of the Study

In this cross-sectional study, we consider sMCI and mMCI samples as the snapshots of underlying pathological process at different progression stages. We attempt to reconstruct their natural sequence with the conjunction/disjunction analysis supplemented by a comparative analysis of our findings with the relevant longitudinal studies of MCI and AD cohorts. Although our conclusion about sMCI to mMCI to AD as a typical progression path agrees with the data from available longitudinal studies, this requires direct confirmation through longitudinal research. Furthermore, the relevance of amnestic MCI condition of our subjects to the underlying AD is supported by one of the early biomarkers – i.e., by the significant reduction of the hippocampal volume – which effectively predicts AD diagnosis and progression [109], [110], [67], as well as correlates with the burden of neuro-fibrillary tangles [111], [112], [113]. However, hippocampal atrophy does not reliably differentiate AD from frontotemporal dementia [114]. The imaging of WM may have better specificity. This calls for further studies comparing the 3D patterns of demyelination across the various neurodegenerative diseases of old age. On the other hand, the relationship between demyelination spread and non-structural biological features of AD should be detailed. Specifically, our findings can be refined further based on a strict sample selection due to the application of the new criteria including biological evidence of AD pathology for aMCI patients and its absence for control participants.

## Supporting Information

Figure S1 **Statistical parametric map of dependence between episodic memory performance and demyelination.** Brain regions with a significant positive voxel-wise dependence (*P*<.05, FWE corrected) between MTR and the delayed cued recall in the RI-48 test for 69 subjects are rendered in different colors corresponding to different anatomical structures. The involved parts of the posterior thalamic radiation (cyan) and of the fornix (violet) are shown in the coronal view. For the posterior thalamic radiation, the average linear slope is.11 and the average R^2^ is.12. For the fornix, the average linear slope is.13 and the R^2^ is.10. For other designations see Fig. 2.
(TIF)

Table S1 Neuropsychological memory scores of amnestic MCI and control subjects. Columns present group characteristics (mean ± standard error, “n” stands for a number of subjects). Statistical comparisons are reported as for Table 1. “NS” stands for “not significant” (*P*≥.05). *refers to *sMCI>mMCI*, **, *controls>sMCI*, and ***, *controls>mMCI*.
(DOCX)

Table S2 Neuropsychological executive scores of aMCI and control subjects. * refers to sMCI vs. executive mMCI, ** sMCI vs. controls, and *** executive mMCI vs. controls. For other designations see Tables 1 and S1.
(DOCX)

Table S3 Demyelination in amnestic MCI patients: Conjunction/Disjunction effects. The table shows the number of voxels with significant changes of MTR (*P*<.05, FWE corrected) and their percentage relative to the total number of voxels spanning respective anatomical structure based on the AAL atlas [62] and the ICBM DTI-81 atlas [63]. Anatomical structures with the size of affected volume ≥1% are included. For other designations see Tables 1 and S1.
(DOCX)

Table S4 Effect size of MTR decrease common to sMCI and mMCI. The multivariate effect size *D* (Mahalanobis distance, see Section 2.4.) and 95% confidence interval (CI) are reported for the conjunction effect in WM and GM. The lower end of all confidence intervals is larger than 1, showing that all effect sizes are large. Given their overlapping CI, all effect sizes are of similar magnitudes. “LH” stands for the left hemisphere, “RH,” for the right hemisphere.
(DOCX)

Table S5 Classification of amnestic MCI. For each binary classification (rows) and brain ROI (columns), three parameters are reported: BA, the mean balanced accuracy (mean of the probability distribution of the balanced accuracy); CI, the confidence interval (95% of mass of the probability distribution of the balanced accuracy); *P*, the *P*-value of falsely rejecting a chance level performance. BA and CI are reported as a percentage.
(DOCX)

Table S6 Multiple linear regression analysis: Episodic memory performance vs. demyelination of white matter. The table shows the number of voxels with significant positive dependence of MTR values on delayed cued recall scores of the RI-48 test (*P*<.05, FWE corrected) and their percentage relative to the total number of voxels spanning the respective anatomical structure. For other designations see Tables S1 and S3.
(DOCX)

Text S1 **Classification of amnestic MCI subjects.**
(DOCX)
