# Supplementary figures and images for: Demyelination in Mild Cognitive Impairment Suggests Progression Path to Alzheimer’s Disease
Source: PLoS One. 2013 Aug 30;8(8):e72759. doi: 10.1371/journal.pone.0072759 (PMC3758332; doi:10.1371/journal.pone.0072759)

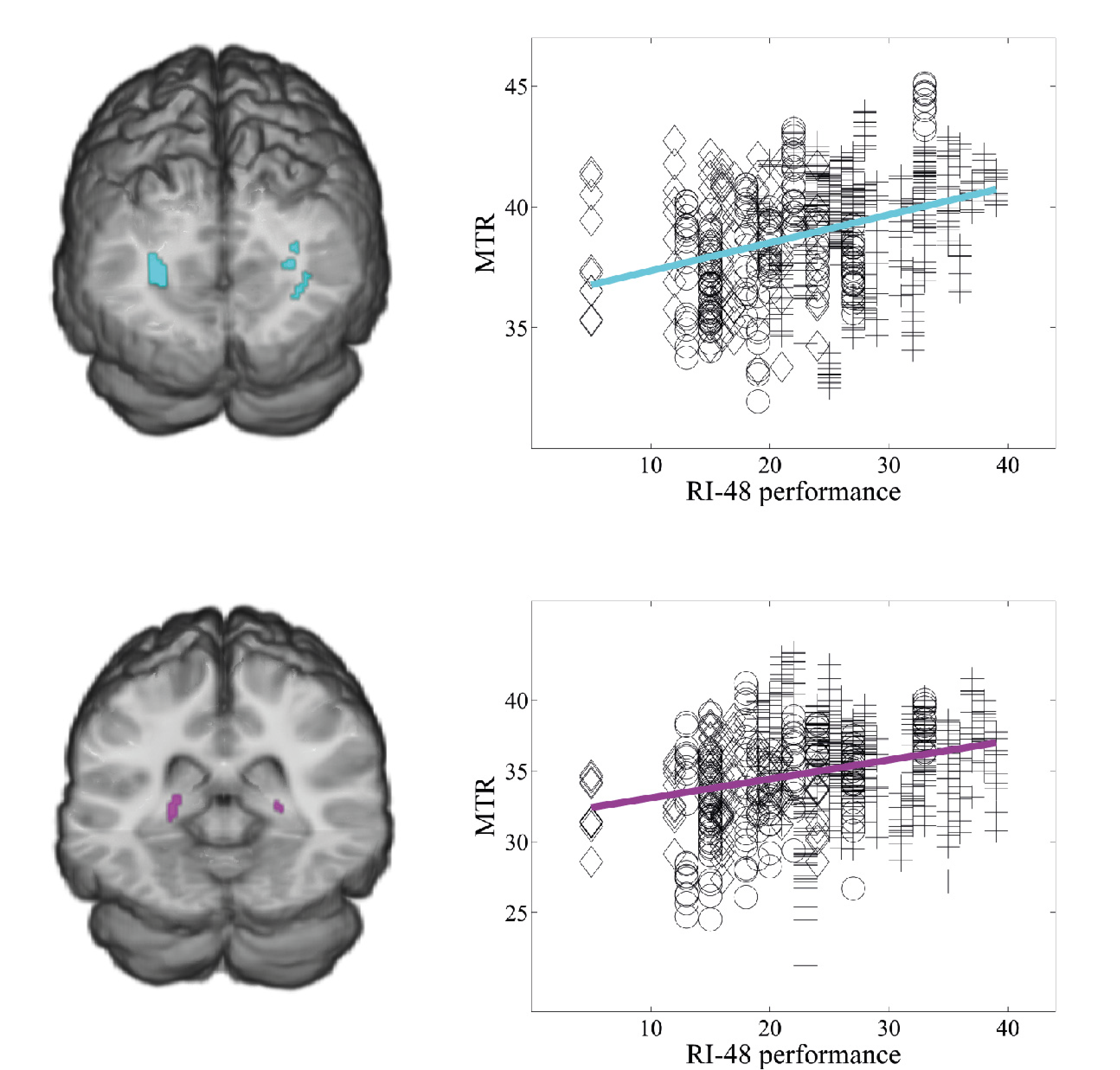

Supplement: Figure S1 — Statistical parametric map of dependence between episodic memory performance and demyelination. Brain regions with a significant positive voxel-wise dependence (P<.05, FWE corrected) between MTR and the delayed cued recall in the RI-48 test for 69 subjects are rendered in different colors corresponding to different anatomical structures. The involved parts of the posterior thalamic radiation (cyan) and of the fornix (violet) are shown in the coronal view. For the posterior thalamic radiation, the average linear slope is.11 and the average R2 is.12. For the fornix, the average linear slope is.13 and the R2 is.10. For other designations see Fig. 2. (TIF) [file pone.0072759.s001.tif]
