# Supplementary material for: Demyelination in Mild Cognitive Impairment Suggests Progression Path to Alzheimer’s Disease
Source: PLoS One. 2013 Aug 30;8(8):e72759. doi: 10.1371/journal.pone.0072759 (PMC3758332; doi:10.1371/journal.pone.0072759)
Supplement: Table S1 — Neuropsychological memory scores of amnestic MCI and control subjects. Columns present group characteristics (mean ± standard error, “n” stands for a number of subjects). Statistical comparisons are reported as for Table 1. “NS” stands for “not significant” (P≥.05). *refers to sMCI>mMCI, **, controls>sMCI, and ***, controls>mMCI. (DOCX) [file pone.0072759.s002.docx]

| **Test** | **Single domain MCI** | **Multiple domain MCI** | **Control** | **Statistical comparisons** |
| --- | --- | --- | --- | --- |
| **RI-48 immediate cued recall max score=48** | 36.2±1.8 (n=17) | 35.9±1.4 (n=10) | 42.3±.5 (n=42) | *NS ***P*<.001 ****P*<.0005 |
| **RI-48**  **delayed cued recall max score=48** | 19.9±1.9 (n=17) | 16.2±1.5 (n=10) | 28.0±.8 (n=42) | *NS ***P*<.0005, ****P*<.0005 |
| **RL/RI 16**  **delayed free recall max score=16** | 7.6±1.5 (n=7) | 4.3±1.0 (n=12) | ― | *NS ― ― |
| **RL/RI 16 delayed total recall max score=16** | 14.1±.6 (n=7) | 10.7±1.3 (n=12) | ― | *NS ― ― |
