# Supplementary material for: Demyelination in Mild Cognitive Impairment Suggests Progression Path to Alzheimer’s Disease
Source: PLoS One. 2013 Aug 30;8(8):e72759. doi: 10.1371/journal.pone.0072759 (PMC3758332; doi:10.1371/journal.pone.0072759)
Supplement: Table S2 — Neuropsychological executive scores of aMCI and control subjects. * refers to sMCI vs. executive mMCI, ** sMCI vs. controls, and *** executive mMCI vs. controls. For other designations see Tables 1 and S1. (DOCX) [file pone.0072759.s003.docx]

| **Test** | **sMCI** | **mMCI** | **Executive mMCI** | **Controls** | **Statistical comparisons** |
| --- | --- | --- | --- | --- | --- |
| **Verbal fluency/ categorical productions (#)** | 27.9±1.4 (n=21) | 20.3±1.6 (n=21) | 20.6±1.9 (n=16) | 32.8±1.0 (n=42) | **P*<.01 ***P*<.05 ****P*<.001 |
| **Verbal fluency/ litteral productions (#)** | 21.9±1.4 (n=21) | 18.5±1.4 (n=21) | 17.3±1.6 (n=16) | 24.5±.9 (n=42) | *NS **NS ****P*<.005 |
| **Trail making/ reaction time (s)** | 108.1±8.3 (n=20) | 174.3±21.3 (n=20) | 199.6±21.4 (n=15) | 85.3±4.6 (n=42) | **P*<.001 **P<.05 ****P*<.0005 |
| **Long Stroop/ reaction time (s)** | 124±6.8 (n=7) | 158.3±14.7 (n=13) | 168.0±15.5 (n=11) | 111.2±9.8 (n=12) | *NS **NS ****P*<.05 |
| **Short Stroop/ reaction time (s)** | 30.4±2.4 (n=14) | 52.0±8.5 (n=8) | 63.4±10.6 (n=5) | 25.6±.9 (n=30) | **P*<.005 **NS ****P*<.0005 |
