# Supplementary material for: Demyelination in Mild Cognitive Impairment Suggests Progression Path to Alzheimer’s Disease
Source: PLoS One. 2013 Aug 30;8(8):e72759. doi: 10.1371/journal.pone.0072759 (PMC3758332; doi:10.1371/journal.pone.0072759)
Supplement: Table S3 — Demyelination in amnestic MCI patients: Conjunction/Disjunction effects. The table shows the number of voxels with significant changes of MTR (P<.05, FWE corrected) and their percentage relative to the total number of voxels spanning respective anatomical structure based on the AAL atlas [62] and the ICBM DTI-81 atlas [63]. Anatomical structures with the size of affected volume ≥1% are included. For other designations see Tables 1 and S1. (DOCX) [file pone.0072759.s004.docx]

| Anatomical Structure | Left Hemisphere | Right Hemisphere |
| --- | --- | --- |
| **White matter - Conjunction** | | |
| **Splenium** | 596 voxels (37%) | |
| **Posterior Corona Radiata** | 7 voxels (2%) | 70 voxels (16%) |
| **Posterior Thalamic Radiation** | NS | 5 voxels (1%) |
| **Gray matter - Conjunction** | | |
| **Parahippocampal Gyrus** | 129 voxels (13%) | 101 voxels (9%) |
| **Hippocampus** | 139 voxels (15%) | 81 voxels (8%) |
| **Lingual Gyrus** | 64 voxels (3%) | 33 voxels (1%) |
| **Fusiform gyrus** | NS | 25 voxels (1%) |
| **Thalamus** | 27 voxels (2%) | 15 voxels (1%) |
| **White matter - Disjunction** | | |
| **Inferior Frontal Gyrus - Pars Triangularis** | NS | 145 voxels (54%) |
| **Middle Frontal Gyrus** | NS | 51 voxels (8%) |
| **Gray matter - Disjunction** | | |
| **Insula** | NS | 100 voxels (6%) |
