# Supplementary material for: Demyelination in Mild Cognitive Impairment Suggests Progression Path to Alzheimer’s Disease
Source: PLoS One. 2013 Aug 30;8(8):e72759. doi: 10.1371/journal.pone.0072759 (PMC3758332; doi:10.1371/journal.pone.0072759)
Supplement: Table S4 — Effect size of MTR decrease common to sMCI and mMCI. The multivariate effect size D (Mahalanobis distance, see Section 2.4.) and 95% confidence interval (CI) are reported for the conjunction effect in WM and GM. The lower end of all confidence intervals is larger than 1, showing that all effect sizes are large. Given their overlapping CI, all effect sizes are of similar magnitudes. “LH” stands for the left hemisphere, “RH,” for the right hemisphere. (DOCX) [file pone.0072759.s005.docx]

| **ROI** | **Cluster-wise effect size** | | |
| --- | --- | --- | --- |
|  | **Volume in voxels (1 voxel = 8mm^3^)** | ***D*** | **CI** |
| **WM/(LH+RH)** | 678 | 4.3 | 2.7-6.8 |
| **GM/LH GM/RH** | 367 255 | 3.2 2.1 | 1.9-4.3 1.2-2.6 |
