# Supplementary material for: Demyelination in Mild Cognitive Impairment Suggests Progression Path to Alzheimer’s Disease
Source: PLoS One. 2013 Aug 30;8(8):e72759. doi: 10.1371/journal.pone.0072759 (PMC3758332; doi:10.1371/journal.pone.0072759)
Supplement: Table S5 — Classification of amnestic MCI. For each binary classification (rows) and brain ROI (columns), three parameters are reported: BA, the mean balanced accuracy (mean of the probability distribution of the balanced accuracy); CI, the confidence interval (95% of mass of the probability distribution of the balanced accuracy); P, the P-value of falsely rejecting a chance level performance. BA and CI are reported as a percentage. (DOCX) [file pone.0072759.s006.docx]

| **Classification/ROI** | **WM** | **GM** |
| --- | --- | --- |
| **Controls vs. sMCI** | BA=54.9, CI=43.8-66.6 *P*=.20 | BA=49.4, CI=40.0-60.8 *P*=.55 |
| **Controls vs. Executive mMCI** | **BA=71.6, CI=59.9-83.3 *P*<.0001** | **BA=71.1, CI=60.2-82.3 *P*<.000001** |
| **sMCI vs. Executive mMCI** | BA=61.3, CI=47.4-75.0 *P*=.055 | BA=61.3, CI=47.4-75.0 *P*=.055 |
