# Supplementary material for: Demyelination in Mild Cognitive Impairment Suggests Progression Path to Alzheimer’s Disease
Source: PLoS One. 2013 Aug 30;8(8):e72759. doi: 10.1371/journal.pone.0072759 (PMC3758332; doi:10.1371/journal.pone.0072759)
Supplement: Table S6 — Multiple linear regression analysis: Episodic memory performance vs. demyelination of white matter. The table shows the number of voxels with significant positive dependence of MTR values on delayed cued recall scores of the RI-48 test (P<.05, FWE corrected) and their percentage relative to the total number of voxels spanning the respective anatomical structure. For other designations see Tables S1 and S3. (DOCX) [file pone.0072759.s007.docx]

| Anatomical Structure | Left Hemisphere | Right Hemisphere |
| --- | --- | --- |
| **Splenium** | 177 voxels (11%) | |
| **Superior Longitudinal Fasc.** | 36 voxels (4%) | 121 voxels (15%) |
| **Posterior Thalamic Radiation** | 58 voxels (12%) | 33 voxels (7%) |
| **Fornix** | 21 voxels (15%) | 7 voxels (6%) |
| **Inf. Longitudinal & Inf. Fronto-occipital Fasc.** | NS | 40 voxels (14%) |
| **Posterior Corona Radiata** | 40 voxels (9%) | 79 voxels (17%) |
| **Precuneus** | NS | 133 voxels (4%) |
| **Hippocampus** | 13 voxels (1%) | 14 voxels (1%) |
| **Middle Temporal Gyrus** | 48 voxels (1%) | NS |
| **Rolandic Operculum** | NS | 26 voxels (2%) |
| **Parahippocampal Gyrus** | NS | 20 voxels (2%) |
