# Supplementary material for: Demyelination in Mild Cognitive Impairment Suggests Progression Path to Alzheimer’s Disease
Source: PLoS One. 2013 Aug 30;8(8):e72759. doi: 10.1371/journal.pone.0072759 (PMC3758332; doi:10.1371/journal.pone.0072759)
Supplement: Text S1 — Classification of amnestic MCI subjects. (DOCX) [file pone.0072759.s008.docx]

***Classification of amnestic MCI subjects.*** To evaluate the performance of MTR-based maps for discrimination between the sMCI, the executive mMCI, and the controls, we applied a classification algorithm to the three pair-wise comparisons of interest: *controls vs. sMCI,* *sMCI vs. executive mMCI,* and *controls vs. executive mMCI*. The algorithm was based on a combined feature selection and linear discriminant analysis (LDA) approach [1]. First, we computed correlation-adjusted *t*-scores from the WM, sWM, or GM masked MTR images. These scores enable an effective ranking of voxels in the presence of correlations among them. Second, we computed false discovery rates (*lfdr*) from the distribution of *t*-scores, and kept only voxels with *lfdr* < .8 [1]. Finally, the LDA classifier was trained on the selected sub-set of voxels by employing James-Stein shrinkage rules in order to cope with the limited number of samples (subjects) compared to the large number of predictors (voxels). This shrinkage discriminant procedure is implemented in the R package sda (http://cran.r-project.org/web/packages/sda).

The accuracy of the binary classifications was assessed by leave-one-out cross-validation. Namely, for each comparison of interest, respectively 63 (42 controls and 21 sMCI), 37 (21 sMCI and 16 executive mMCI), and 58 (42 controls and 16 executive mMCI) partitions were created, each of them having one subject for a test image and the remaining ones for learning images. Since the sizes of classes were different, we computed the balanced accuracy [2]. Within a Bayesian framework of classification generalizability, uncertainty (or confidence intervals) of the balanced accuracy can be computed by modeling the posterior distribution with a beta distribution [2]. To determine whether the classification was above chance level, a *P*-value from that distribution was estimated as well.

**References**

1. Ahdesmäki M, Strimmer K (2010) Feature selection in omics prediction problems using cat scores and false non discovery rate control. The Annals of Applied Statistics 4: 503-519.

2. Brodersen KH, Soon Ong C, Stephan KE, Buhmann JM (2010) The balanced accuracy and its posterior distribution. 20^th^ International Conference on Pattern Recognition, Istanbul.
